# Antenatal Food Avoidances in Madagascar Suggest an Evolutionary Link Between Subsistence Patterns, Carbohydrate Consumption, and Determinants of Obstructed Labor

**DOI:** 10.1002/ajpa.70029

**Published:** 2025-03-19

**Authors:** Ornella Maggiulli, Jean Freddy Ranaivoarisoa, Hary Raliarison, Fifaliana Andriamanantena, Sabrinah Raherimamonjy, Mikanto Rabearison, Jay T. Stock, Jonathan C. K. Wells

**Affiliations:** ^1^ Childhood Nutrition Research Centre, Population, Policy and Practice Research and Teaching Department UCL Great Ormond Street Institute of Child Health London UK; ^2^ Département de Paléontologie et d'Anthropologie Biologique University of Antananarivo Antananarivo Madagascar; ^3^ Department of Anthropology, Social Science Centre Western University London Ontario Canada

**Keywords:** agriculture, food avoidances, Madagascar, obstetric dilemma, obstructed labor, pregnancy

## Abstract

**Objectives:**

We aimed to test if different subsistence patterns shaped different antenatal eating behaviors in Madagascar, and to investigate if reasons given for maternal dietary restrictions disclosed actual biological pressures on pregnancy.

**Material and Methods:**

We conducted semi‐structured interviews with 312 participants to investigate differences in avoided food types during pregnancy, reasons, and infants' weight between subsistence patterns (agriculture, agriculture‐husbandry/fishery, fishery), and associations between food types and reasons (Chi‐squared, Fisher's, and Kruskal–Wallis test in R and SPSS). Secondary questions investigated regional variance in food avoidance (PCA), the association between the carbohydrate content of avoided foods and the fear of difficult delivery (regression analysis), and institutional and non‐institutional influences on dietary proscriptions (heatmap).

**Results:**

Agriculturalists avoided more plant‐based foods than fishers for the fear of difficult delivery due to large infants. Infants' weights at birth did not vary significantly across subsistence modes. Dietary norms were reinforced by an interplay between institutional and non‐institutional advisors.

**Conclusions:**

Food avoidances during pregnancy among Malagasy agriculturalists and fishers differ in targets and reasons. Avoided foods reflect staple diets, while the fear of difficult labor due to large infant size in relation to carbohydrate‐rich foods among agriculturalists overlaps with a high incidence of obstructed labor in agricultural regions. Therefore, different subsistence modes affect antenatal behavior priorities differently. Taboos and sources of advice on maternal diet are fluid systems. We highlight the urgent need to better understand the determinants of obstructed labor and the patterns of spread of antenatal practices in Madagascar.

## 1 Introduction

In Madagascar, taboos are omnipresent in the regulation of daily activities and social behavior (Ruud 1960; Golden and Comaroff 2015). Malagasy taboos, or *fady* in the local language, target actions, objects, words, and places that are perceived to be antithetical to a given individual or collective purpose, usually during specific life events (Ruud 1960). The most characteristic prohibitions are those that address pregnancy, childbirth, and childhood (Poirier 1978), a scenario that seems to be a general trend among cross‐cultural taboos (Meyer‐Rochow 2009). Gestation and childbirth are the most sensitive physiological periods during development and are exposed to a pool of pivotal selective pressures in evolutionary terms (Brown et al. 2013). Therefore, the fact that reproductive stages are cross‐cultural targets of nutritional restrictions opens unexplored opportunities for research in the evolution of human culture and health.

In a recent meta‐analysis of existing literature on cross‐cultural food taboos during pregnancy, Maggiulli et al. (2022) highlighted that the majority of food taboos during pregnancy in agricultural societies seem to target carbohydrates due to the fear of difficult delivery and that this pattern contrasts with taboos in non‐agricultural societies. This has been interpreted in light of the hypothesis that recent nutrition transitions have exacerbated the human difficulty in giving birth by enlarging infant size, while in some periods also decreasing the stature of females (Maggiulli et al. 2022; Wells et al. 2012). There has been substantial debate over the human difficulty in giving birth since Washburn (1960) proposed the antagonistic roles of bipedalism and childbirth in shaping the female pelvis in the “obstetrical dilemma” hypothesis. Currently, there is a general trend towards more nuanced interpretations of this hypothesis, acknowledging both multiple factors and trade‐offs and local manifestations, some of them reflecting plasticity (Wells et al. 2012; Haeusler et al. 2021; Grunstra et al. 2023; Warrener 2023). However, in‐depth studies of individual populations are needed to outline the determinants of dietary attitudes as well as perceived and real health threats during pregnancy before inferring robust evolutionary models to explain patterns of dietary behavior and propose healthcare strategies.

The main goal of the present work was to evaluate evolutionary cues in cultural norms, rather than focusing on any physiologic responses relating to food aversions or cravings. The hypothesis that food aversions are evolved mechanisms to protect the fetus from harmful factors is supported by Mckerracher et al. (2015, 2016) in the Fijian islands but not by Placek and Hagen (2015) in India. While Maggiulli et al. (2022) described a first attempt to analyze food taboos during pregnancy cross‐culturally and systematically, a similar cross‐cultural and systematic analysis of food aversions seems to currently be lacking in the literature, preventing any robust evolutionary interpretation both of food aversions alone and of their relation to taboos. For these reasons, in this study we analyzed only culturally reinforced food avoidances, namely taboos, in Madagascar and not personal food aversions.

The study of antenatal dietary behavior in Madagascar provides a useful test of the relationship between cultural norms and health threats because a rooted system of prohibitions that affects maternal diet combines with strong social pressure for women to become mothers (Pourette et al. 2018) and with a high burden of severe clinical complications. Madagascar has a maternal mortality rate of 426 per 100,000 live births and a neonatal mortality rate of 22‰ (Institut National de Statistique (INSTAT) and United Nations of International Children's Emergency Fund (UNICEF) 2018). With 60% of births taking place at home (Institut National de Statistique (INSTAT) and United Nations of International Children's Emergency Fund (UNICEF) 2018), it is not easy to obtain a comprehensive record of actual birth complications in Madagascar. However, a survey on the causes of maternal death in hospitals and rural health care facilities revealed that the principal causes of maternal mortality are hemorrhage (19.5%), complications after miscarriage (16.0%), eclampsia and pre‐eclampsia (12.5%), uterine rupture (8.2%) and prolonged labor/dystocia (7.6%) (Vice Primatrue Charge de Sante Publique et al. 2010). Rates of all‐cause maternal mortality appear not to differ between hospitals and rural facilities, except for dystocia, which increases substantially to 42.9% in rural facilities (Vice Primatrue Charge de Sante Publique et al. 2010). Moreover, the assistance provided by qualified health staff covers 72% of births in urban contexts but decreases to 39% in rural areas. In contrast, *renizaza* (traditional birth attenders) assist 23% of childbirths in urban contexts and 50% in rural areas.

High rates of dystocia are consistent with the widespread concern over “big babies” and difficult delivery, against which food restrictions to limit the size of the fetus are common (Pourette et al. 2018). Antenatal maternal behavior is influenced not only by elders, *renizaza* (traditional birth attenders) and *ombiasy* (witch‐doctors who live in remote areas of the south and who are consulted during challenging life events), who transmit the will of the ancestors, but also by a fragile net of institutional healthcare professionals within a frame of “medical pluralism” which remains understudied (Pourette et al. 2018; Andrianantoandro et al. 2021). The main modes of food supply in Madagascar (agriculture and fishing) provide contrasting availability of carbohydrates across geographical regions, resulting in high carbohydrate consumption in the farming highlands but severe scarcity on the fishers' coast (Institut National de la Statistique de Madagascar 2010; Dostie et al. 2002). However, the associations of this subsistence variability with maternal dietary behavior and pregnancy outcomes have not been explored.

The present study is a novel attempt to combine original ethnographic data in Madagascar with demographic data available in the literature in order to investigate associations of foods avoided during pregnancy with the perception of risks associated with their consumption across the diversity of subsistence strategies and related staple diets.

The study had four main aims:

- To test whether there are significant differences in food types avoided during pregnancy between communities whose subsistence is based on (a) agriculture (b) agriculture and animal husbandry, and (c) fishing.
- To test for significant differences in clusters of reasons for food avoidance (to be identified a posteriori after data collection) between subsistence modes.
- To test whether food types were associated with specific clusters of reasons for food avoidance.
- To test for differences in infant birth weight, as reported by mothers, between subsistence modes.

In addition, our secondary aims were to explore (a) the relation between the carbohydrate content of each reported food and its avoidance for fear of large babies, (b) which variables explained regional variation, and (c) the convergence of institutional and non‐institutional sources of advice regarding avoidance of each reported food.

## 2 Materials and Methods

### 2.1 Data Collection

Data collection on food avoidances during pregnancy was conducted from May to July 2018 in collaboration with the University of Antananarivo, Department of Paleontology and Biological Anthropology. A sample of 312 women who experienced at least one pregnancy were interviewed in 4 different regions of Madagascar: Highlands (central Madagascar), Toliary (southwestern coast), Marovoay (Northwest) and Maroantsetra (Northeast). The regions to survey were selected according to the different subsistence patterns typical of the area, namely agriculture alone (Highlands, Marovoay, Maroantsetra), agriculture in combination with minor local subsistence modes, such as animal husbandry (Highlands, Marovoay) or fishery (Maroantsetra) termed agriculture‐husbandry/fishery, and fishery alone (Toliary, Maroantsetra). Table 1 shows the sample size and subsistence pattern distribution for each region.

**TABLE 1:** Sampled communes stratified by main area, region and district, indicating main subsistence mode and number of respondents.

Region	District	Commune	*N*	Percent of interviewed women practicing different subsistence modes in each region
Highlands Central Madagascar	Itasy (pilot study)	Ilot de la Vierge	1	69.9% agriculture‐husbandry, 28.2% agriculture, 1.9% (lake) fishery
		Ampefy	2	
		Sahampetraka	3	
		Ifanja	1	
		Tranotelo	1	
		Skonondrano	1	
		Ampahimanga	8	
	Vakinakaratra	Ambohibary	17	
		Behenjy	5	
		Ambatolampy	19	
		Analovary	1	
		Ansitrabe	1	
	Amoron'i Mania	Ambositra	22	
		Fandriana	21	
		Total	103	
Toliary Southwestern coast	Toliary‐II	Ifaty	14	98.8% (marine) fishery, 1.2% agriculture‐fishery
		Amdranomankanga	4	
		Manombo	14	
		St Augustin	15	
		Soalara	17	
		Anakao	17	
		Total	81	
Marovoay Northwestern Madagascar	Marovoay	Marovoay	13	89.5% agriculture, 7.9% agriculture‐husbandry, 2.6% fishery
		Ankarafantsinka	25	
		Total	38	
Maroantsetra Northeastern coast	Maroantsetra	Tanantsaka	30	46.8% agriculture, 39% agriculture‐fishery, 14.3% fishery
		Ambodipaka	18	
		Ambodibary	10	
		Mahasoa	19	
		Total	77	
Total = 299				

The principal investigator (PI) and one female research assistant presented to the principle of the main cities of each selected region a presentation letter from the University of Antananarivo and provided an introductory talk on the project. A local guide introduced the PI and assistant to unrelated households in nearby villages. The team visited 10 to 30 households per village (Table S1) according to population density. The data collection was based on semi‐structured interviews lasting 20 min to 1 h. The structured part of the interview was based on a questionnaire, illustrated to the subject and manually completed by the research assistant, which asked about the following information: main subsistence pattern of the household, general complications feared during pregnancy, complications actually experienced, names of avoided foods, reasons, source of the advice, number of daily meals before and during pregnancy, overall perception of the pregnancy experience, and weigh at birth of children. All information was asked using open questions (e.g., What is the main subsistence pattern of your household?); after asking whether the woman followed any food taboo during pregnancy, she was invited to list the name of the avoided foods together with reasons for the avoidance and the source of advice, and the responses were inserted by the research assistant into a table whose three columns referred to food name, reason and advice source. The questionnaire and interviews were written/conducted in Malagasy, with translation in local dialects if needed. All interviews were back‐translated in French and English to the PI, who had basic knowledge of Malagasy language. The Comité d'Ethique de la Recherce Biomédicale (CERBM) of the Ministére de la Santé (Madagascar) declared that the present research did not imply biomedical risk (N°: 32 MSANP/SG/AGMED/CERBM). All women gave their consent to participate anonymously to the study. The data that support the findings of this study are available in the Supporting Information of this article.

### 2.2 Exclusion Criteria and Statistical Analysis

Women whose diet did not directly depend on their subsistence strategy, that is, who had occupational activities other than agriculture or fishery (e.g., teachers and retailers), were excluded from analyses. Zebu pastoralists were excluded from the interviews because zebu cattle are mainly raised to maintain high social status and/or wealth rather than for self‐consumption (Hänke and Barkmann 2017).

The main variables extracted from the questionnaires on which the semi‐structured interviews were based were: “Subsistence pattern” of the respondent (agriculture, agriculture‐husbandry/fishery, and fishery); avoided “Food type” (animal product, plant product, and miscellaneous); “Reason focus” for food avoidance (big baby and/or difficult delivery, varied physiologic complications, nonphysiologic complications), which was categorized a posteriori and overlapped the same qualitative gradient identified previously in a cross‐cultural study of reasons for food taboos during pregnancy (Maggiulli et al. 2022); infant birth weight as recalled by mothers. We acknowledge that the latter variable represents a limitation of this study, as mothers may not recall their infant's weight accurately. However, maternal reports were the only information on birth weight available at the time of the survey, due to the lack of official documentation released by birth attendants. Table S1 shows the full list of variables used in this study and their coding style.

We prepared and coded the data using SPSS (version 27) (Corp 2020), following the guidelines reported in Supporting Information. We then imported contingency tables obtained in SPSS into RStudio (RStudio Team 2021; packages: *base*, *datasets*, *graphics*, *grDevices*, *methods*, *stats*, *utils*, *dplyr*) to run the following statistical and graphical analyses to test our four main hypotheses. The cut‐off for statistical significance was *p* < 0.05.

- Chi‐squared test was used to investigate significant differences in food types avoided during pregnancy between subsistence patterns. The residuals of the independence tests were analyzed and interpreted through mosaic plots.
- Fisher's Exact test was used to investigate significant differences in the focuses of reasons for the food avoidance between subsistence patterns. The residuals of the independence tests were analyzed and interpreted through mosaic plots.
- Fisher's Exact test was used to investigate whether food types were associated with specific focuses of reasons for food avoidance. The residuals of the independence tests were analyzed and interpreted through mosaic plots.
- Kruskal–Wallis nonparametric test was used to test for significant differences in infants' weight at birth, as reported by mothers between subsistence modes. We used a nonparametric test because a previous Shapiro–Wilk test on data did not confirm normality within groups (*p* < 0.001).

We then investigated the following three secondary questions.

- We used linear regression to test if the percentage carbohydrate content of avoided food (Charrondière et al. 2020) was associated with the percentage of women who avoided a given food for the fear of increased birth weight and difficult delivery.
- We used principal component analysis (PCA) to visualize differences in the following proportions between the four surveyed regions: the proportion of women who avoid at least one food in each region, based on the total number of women surveyed in that region (hereafter referred to as avoiders); the proportion of agriculturalists and fishers in each region, based on the total number of women surveyed in that region; the proportion of food avoidances explained by difficult delivery/varied physiologic complications/non‐physiologic in each region, based on the total number of taboos cited in that region; and the proportion of plant/animal/miscellaneous foods avoided in each region, based on the total number of taboos cited in that region.
- We visualized convergence between institutional (hospital staff) and non‐institutional (elders, *renizaza*, *ombiasy*) advice on the avoidance of each reported food using a heatmap.

## 3 Results

### 3.1 Overview

Of 312 questionnaires collected, 299 met the inclusion criteria. Women that were solely agriculturalists represented 33% of the sample, those practicing agriculture‐husbandry/fishery 36%, and fishers 31%. 100 respondents did not report any avoidances during pregnancy. The remaining 199 reported a total of 377 taboos referring to animal (14%), plant (40%) or miscellaneous products (46%) respectively (Table S2). The reasons given for avoidance are reported in Table S3 and stratified by the focus of avoidance. In this and the following tables showing reasons given for food avoidance, the Malagasy term *albumina* indicates an excess of salt in the blood and the swelling of legs or of the uterine cervix, which would obstruct the passage of the infant during delivery; the term *farasisa* indicates diseases that cause skin rashes.

The focus of avoidance mirrored the qualitative gradient in detail and physiologic likelihood, previously identified in cross‐cultural food taboos during pregnancy (Maggiulli et al. 2022). A group of avoidances (5%) was not, or not obviously, linked to specific or plausible physiologic dangers for mothers and infants, and includes fears that the characteristics of the food may transfer to the mother or child and general witchcraft. This group is referred to as *Non physiologic complications*, though future research is needed to investigate if the symbolism here ascribed to this category conceals actual health concerns. Other avoidances referred to specific and plausible health risks, but none of these risks were mentioned frequently enough to develop a separate semantic category (*Varied physiologic complications*) (21%), whereas by contrast a consistent group of reasons (74%) referred to *Big baby and/or difficult delivery*, a condition that is highly detailed and specific to childbirth. The mean number of meals self‐reported during pregnancy (2.86 ± 0.621) did not differ from that before or after pregnancy (2.86 ± 0.52). Percentages of low (< 2.5 kg), normal (2.5–3.9 kg) and high (> 3.9 kg) birthweight were 11.1%, 80.9%, and 7.7% respectively (Table S4). Personal fears about pregnancy (Table S5) and self‐reported complications experienced during delivery (Table S6) revealed difficult labor as the most frequently mentioned complication. Among 289 respondents, 78% reported a positive overall perception of the pregnancy experience, while the shares of women who reported a neutral or negative perception were equal (11%).

We illustrate the results related to our main four hypotheses and three secondary questions below in separate subheadings.

### 3.2 Hypothesis I Differences in Food Types Avoided During Pregnancy Between Subsistence Patterns

**FIGURE 1:**
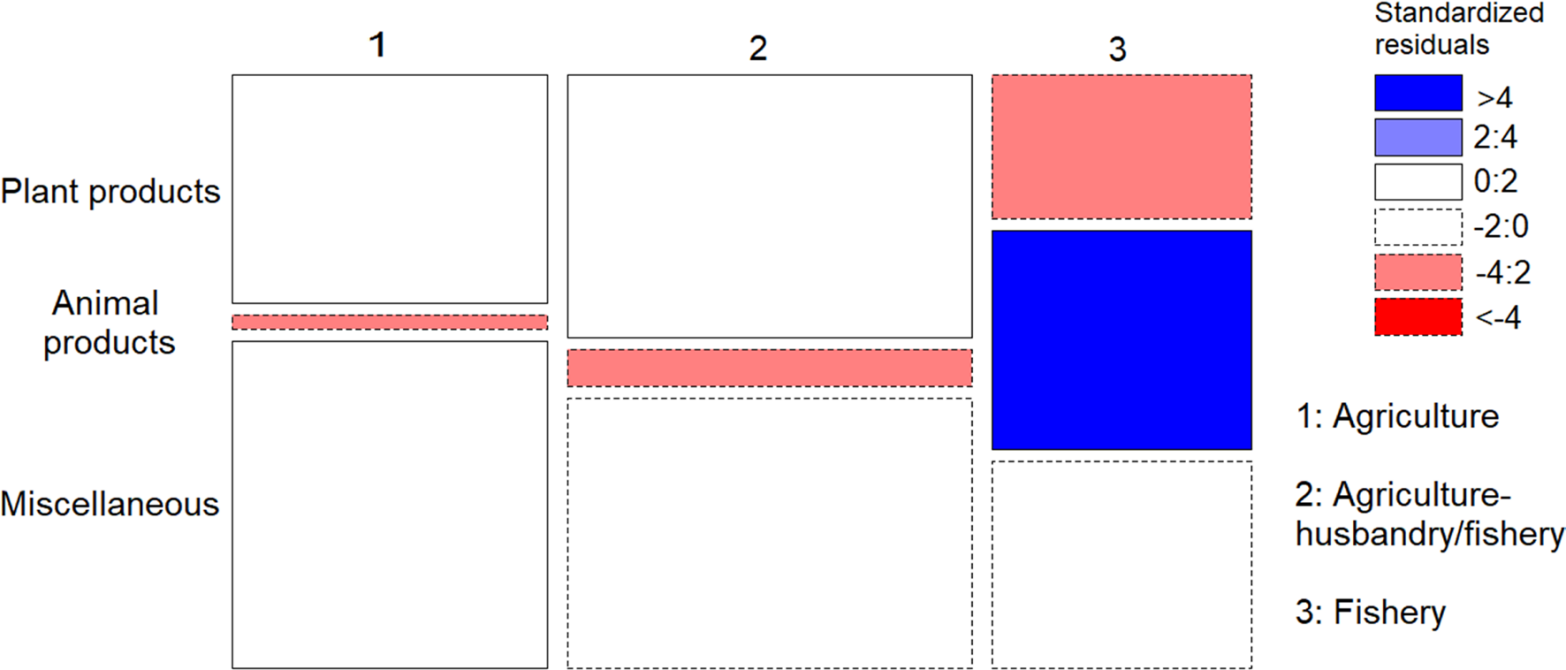
Mosaic plot showing, which cells contribute most to the significance of the test of independence (Chi‐squared) between subsistence patterns and avoided food type. The width of squares represents the numerosity of each subsistent pattern category, while the height represents the numerosity of each food type within each subsistence pattern. Pattern of blue (positive values of standardized Pearson residuals) show cells whose observed frequency is greater than would be found under independence. Pattern of red (negative values) show cells whose observed frequency is lower than would be found under independence.

**FIGURE 2:**
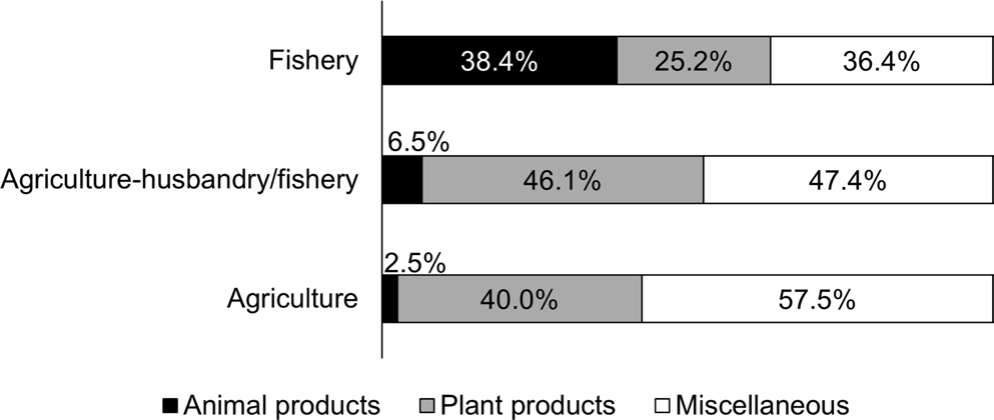
Percentage of animal products (black), plant products (gray), and miscellaneous (white) avoided by respondents practicing agriculture, agriculture‐husbandry/fishery and fishery.

We found significant differences in avoided food types during pregnancy within and between the three subsistence groups (*X* ^2^ = 72.934, df = 4, *p* < 0.001). According to standardized Pearson residuals (Figure 1), in both the agriculture and agriculture‐husbandry/fishery groups, the observations of *Animal food* avoidances were fewer than would be expected under the null model of independence, indicating that these groups were more likely to avoid *Plant products* (40% and 46% respectively) and *Miscellaneous* foods (56% and 47%) than *Animal products* (3% and 7%) (Figure 2). The opposite trend was shown by fishers, who avoided fewer *Plant products* (25%) and more *Animal products* (38%) than expected in the null model (Figures 1 and 2). Figure S1 shows food names avoided by agriculturalists and fishers.

### 3.3 Hypothesis II Differences in Reasons for Food Avoidance Between Subsistence Patterns

**FIGURE 3:**
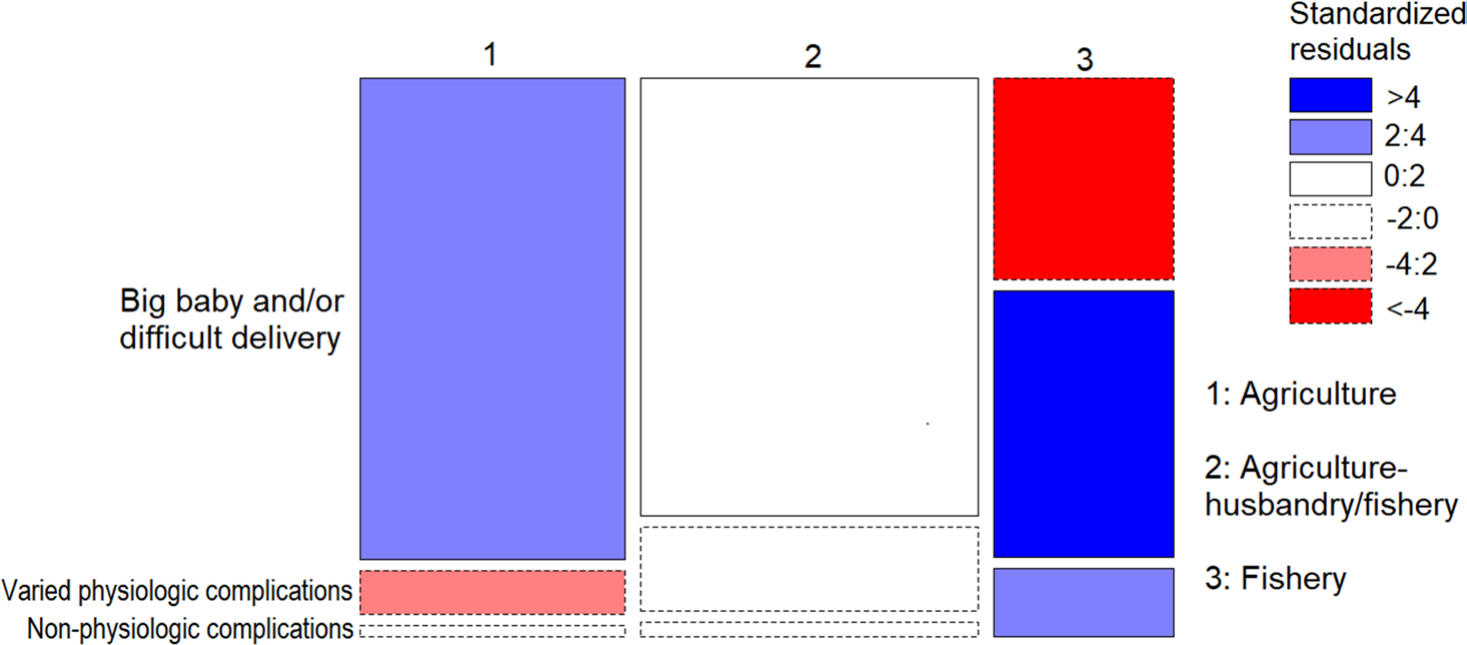
Mosaic plot showing which cells contribute most to the significance of the test of independence (Fisher's Exact test) between subsistence patterns and focus of reason for food avoidance. The width of squares represents the numerosity of each subsistent pattern category, while the height represents the numerosity of each category of reason focus within each subsistence pattern. Pattern of blue (positive values of standardized Pearson residuals) show cells whose observed frequency is greater than would be found under independence. Pattern of red (negative values) show cells whose observed frequency is less than would be found under independence.

**FIGURE 4:**
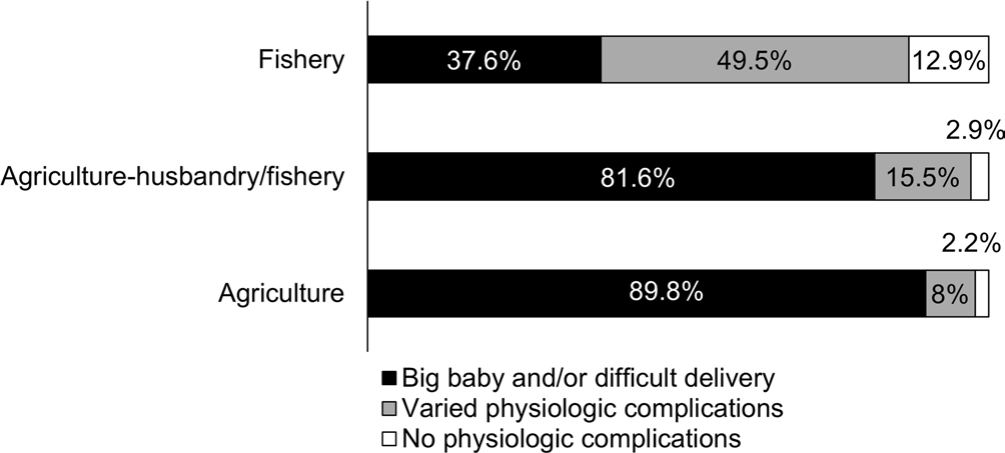
Percentage of reasons for food avoidance referred to big babies and/or difficult delivery (black), varied physiologic complications (gray), and nonphysiologic complications (white) avoided by agriculturalist alone, agriculturalist‐husbandry/fishery and fisher respondents.

The Fisher's Exact test revealed significant differences in the focus of reasons for avoiding foods during pregnancy within and between agriculturalist, agriculture‐husbandry/fishery, and fisher respondents (*p* < 0.001). The observations of *big babies and/or difficult delivery* as a reason for food avoidance among agriculturalists were more frequent than would be expected under the null model of independence, while those of *Varied physiologic complications* were less frequent (Figures 3 and 4). While the results for agriculture‐husbandry/fishery respondents mirrored those for agriculturalists, though with less extreme values of standardized Pearson residuals, fishers showed an opposite pattern, with more marked concern over *Varied or non‐physiologic complications* and *Non physiologic complications*, and a lesser concern over *Big babies and/or difficult delivery* than would be expected under the null model of independence (Figures 3 and 4).

### 3.4 Hypothesis III Association Between Food Types and Reasons for Food Avoidance

**FIGURE 5:**
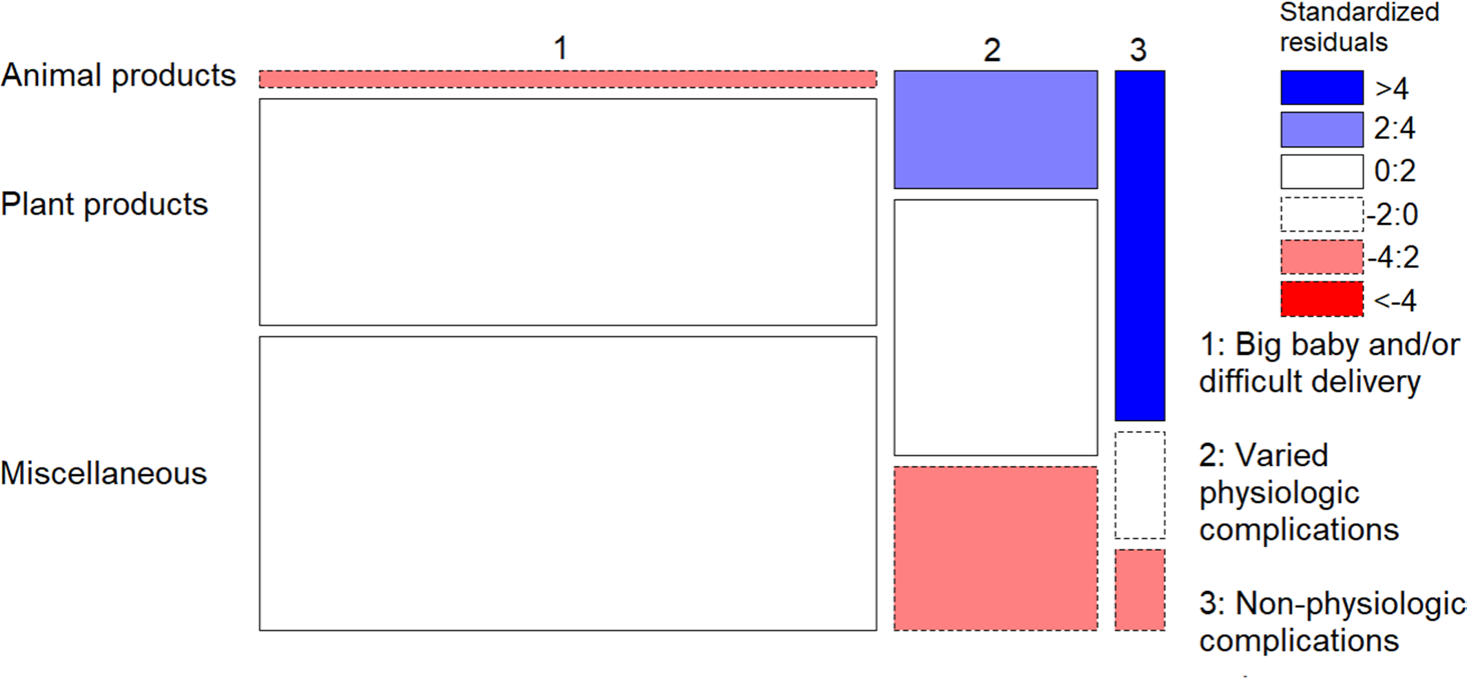
Mosaic plot showing which cells contribute most to the significance of the test of independence (Fisher's Exact test) between focus of the reason for food avoidance and food type. The width of squares represents the numerosity of each category of reason focus, while the height represents the numerosity of each category of food type within each category of reason focus. Pattern of blue (positive values of standardized Pearson residuals) show cells whose observed frequency is greater than would be found under independence. Pattern of red (negative values) show cells whose observed frequency is less than would be found under independence.

**FIGURE 6:**
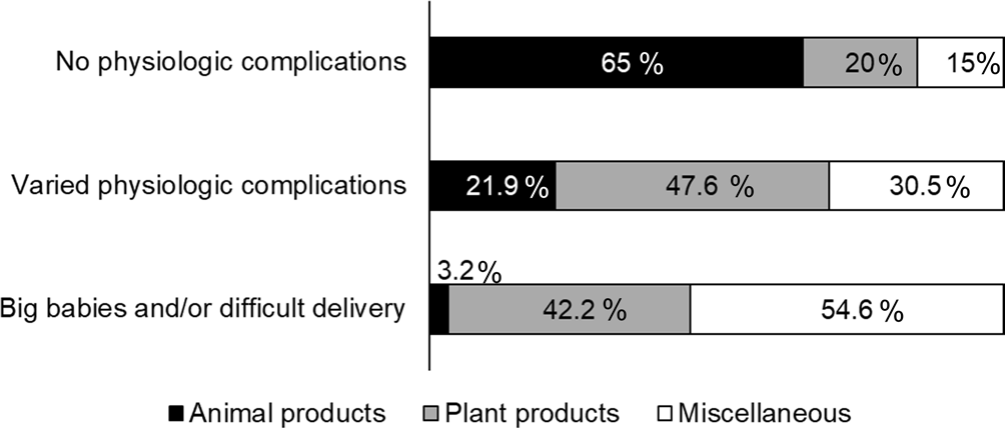
Percentage of animal products (black), plant products (gray), and miscellaneous (white) attributed to big babies and/or difficult delivery, varied physiologic complications and nonphysiologic complications.

The Fisher's Exact test revealed that the distribution of avoided food types differed significantly between categories of focus of the avoidance (*p* < 0.001). The observations of *Animal products* were less frequent than would be expected under the null model of independence as target of fears of *big babies and/or difficult delivery*, which were more likely to be associated with *Plant* and *Miscellaneous* products. By contrast, the observations of *Animal products* as target of *Varied* and *Non‐physiologic complications* were more frequent than would be expected under the null model of independence, while those of *Miscellaneous* were less (Figures 5 and 6). Table 2 shows the frequencies of the association between foods and the detailed reason for the avoidance.

**TABLE 2:** Foods avoided during pregnancy and specificreasonsn for their avoidance grouped by food type and focus of the reason.

	Big babies and/or difficult delivery	Varied physiologic complications	Non physiologic complications
Plant products	Flour (big baby, 49); banana (big baby, 30); peanuts (too much oil for the baby and difficult delivery, 8; big baby); pasta (big baby, 4); bread (big baby, 3); melon (big baby, 2); haninkotrana (big baby, 2); cassava (big baby, 2); avocado (albumina); rice (big baby); yam (big baby); sweet potatoes (big baby)	Peanuts (sickness); pasta (nausea); melon (nausea; sickness; abortion; diarrhea for the baby); cassava (bone decalcification, 2; too thin baby; hemorrhage); rice (nausea); mango (bone decalcification; skin spot on the back); ravitoto (too thin baby, 2; bone decalcification); anantsipolitra (too thin, 6); pepper (farasisa, 3; abortion; skin problems); beans (stomach‐ache, 2; sickness); ginger (sickness; abortion); corn (hemorrhage); sesame (abortion); anana (too thin baby, 2; stomach‐ache); sweet potatoes (nausea); tamarind (too thin baby)	Avocado (dirty baby); pepper (trembling during delivery, 2); sosoa (soft baby)
Animal products	Milk (big baby, 4); eggs (big baby)	Milk (abortion); fish (allergy); animal dead for natural causes (abortion); eggs (handicap); hedgedog (abortion); pig (sickness, 5; abortion, 3; handicap, 2; stomachache; nausea); octopus (itchy for the baby, 3)	Chicken (witchcraft); crab (excessive salivation for the baby, 2); dolphin (witchcraft); duck (webbed fingers, 3); pig (crazy baby, 3; its oil contaminates the baby, 2); moray (witchcraft)
Miscellaneous	Salt (albumina, 85, big baby, 29); oil (albumina, 12; big baby, 3; too much oil for the baby and difficult delivery, 2); sugar and sugary foods (big baby, 3); foods that make the baby bigger (big baby); hot water (big baby)	Salt (farasisa, 13; sickenss); oil (boils); alcohol (farasisa, 3; handicap); spice (farasisa, 2); tambavy (farasisa, 2; yellow fever, 2)	Acid food (trembling during delivery); spices (trembling during delivery): twin foods (twin organs)

*Note:* Frequencies of each reason greater than one are indicated.

### 3.5 Hypothesis IV Differences in Infants' Weight at Birth Between Subsistence Patterns

The average self‐reported birth weight of offspring across the whole sample was 3.17 ± 0.74 kg. We tested for normality of birth weight within groups of women practicing agriculture, agriculture‐husbandry/fishery, and fishery using a Shapiro–Wilk test. Since the test was significant (*p* < 0.001), we rejected normality within groups and ran a Kruskal–Wallis nonparametric test to test for significant differences in infant birth weight between subsistence modes. We did not find significant differences (Chi‐squared = 3.4205, df = 2, *p* = 0.181) between the average weight at birth among agriculture (3.26 ± 0.72 kg), agriculture‐husbandry/fishery (3.06 ± 0.46 kg), and fishery (3.21 ± 0.95 kg) subsistence patterns. Figure S2 shows the boxplot with added jitter to visualize the distribution of weight at birth across groups.

### 3.6 Secondary Question A. Association Between Carbohydrate Content of Avoided Food and Avoidance for the Fear of Large Infants and Difficult Delivery

**FIGURE 7:**
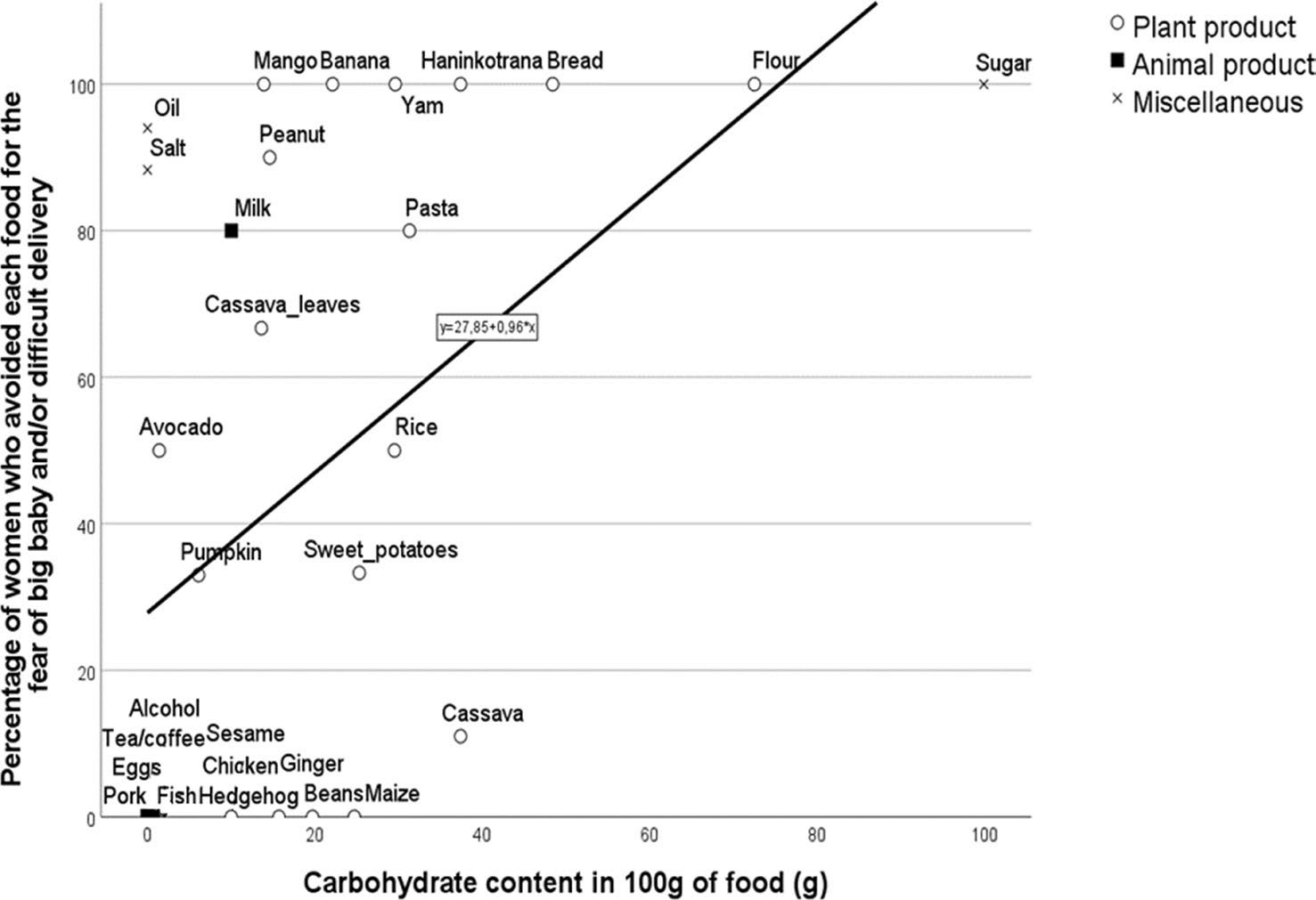
Linear regression plot. The carbohydrate content in 100 g of an avoided food (g) significantly predicted the percentage of women that avoided that food for the fear of increased birthweight and difficult delivery, *y* = 27.850 + 0.956 × (*R* ^2^ = 0.254, *F*(1, 28) = 9.554, *p* = 0.004).

Simple linear regression was used in SPSS to test the association between the carbohydrate content of an avoided food (g) (Charrondière et al. 2020) and the percentage of women that avoided that food for fear of increased birthweight and difficult delivery. The regression model was: Percentage of women = 27.850 + 0.956 × (carbohydrate content). The overall regression was statistically significant (*R* ^2^ = 0.254, *F*(1, 28) = 9.554, *p* = 0.004) (Figure 7).

### 3.7 Secondary Question B. Regional Variance in Antenatal Food Avoidance

**FIGURE 8:**
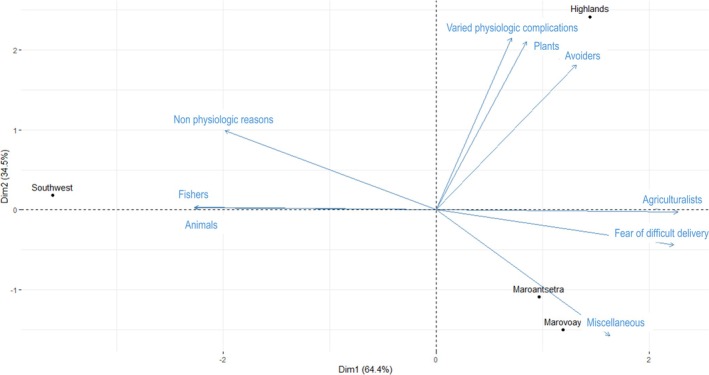
Principal component analysis (PCA) biplot of the location of respondents (Southwest, Highland, Maroantsetra, Marovoay, displayed as dots) and variables about habits in dietary behavior (avoidance of Animal/Plant/Miscellaneous products; reasons focused on Difficult delivery/Varied physiologic complications/Non‐physiologic complications; being Agriculturalists/Fishers; proportion of Avoiders, that is of women who avoided at least one food during pregnancy; displayed as vectors). Regions on the same side as a given variable have a high contribution to it, while the length of the vectors is proportional to their contribution to PC1 and PC2. Positively correlated variables correspond to vectors pointing in similar directions, while negatively correlated ones correspond to vectors pointing in opposite directions.

We investigated if the proportion of women practicing food avoidances and the type of food avoidances varied across regions. The PCA showed that the proportion of avoiders and agriculturalists, the fear of big babies and/or difficult delivery, and varied physiologic complications, as well as the avoidance of plant and miscellaneous products, were a block of positively correlated variables that had higher values in agricultural regions such as highlands, Marovoay, and Maroantsetra. This block of variables was negatively correlated with the presence of fishers and the avoidance of animal food due to non‐physiologic reasons, which had higher values in the southwest coast of Tulear (Figure 8). The PCA loadings and scores are shown in Table S7.

### 3.8 Secondary Question C. Convergence of Institutional and Non‐Institutional Sources of Advice on Avoidance of Food

Frequencies of foods to be avoided and reasons sorted by source of advice (Tables S8 and S9) were used to produce a heatmap (Figure S3). The heatmap shows normalized frequencies of each food taboo within clusters of reasons advised by institutional medical staff and noninstitutional sources. Nonphysiologic and particularly varied physiologic reasons target several foods and are advised against by both non‐institutional sources and institutional medical staff, with no evident convergence of the two sources of advice on the same food taboo. In contrast, salt, flour, and banana were most frequently advised against by both institutional medical staff and non‐institutional sources to avert the delivery of large infants and difficult delivery.

## 4 Discussion

### 4.1 Overview

In this sample of women from households pursuing different subsistence strategies, 100 out of 299 respondents did not report observing any food avoidances during pregnancy, even if they knew that taboos are widespread in Madagascar. This result aligns with the fact that not all communities, families, and individuals strictly adhere to an existing and known system of dietary prohibitions, for multiple reasons that may depend on their lived experience (Golden and Comaroff 2015), such as access to formal education and exposure to food scarcity (Ravaoarisoa, Rakotonirina, et al. 2018). In the present study, the mean number of meals consumed per day during pregnancy did not vary from that before or after pregnancy, converging at 3 meals. However, the food avoidances reported by the majority of 199 women confirmed our first three hypotheses about the differential distribution of food avoidances and reasons between subsistence patterns, but not the fourth hypothesis regarding differential birthweight between agriculturalists and fishers.

### 4.2 Hypothesis I Food Avoidances Mirror Staples of Different Subsistence Patterns

The 199 respondents who followed food avoidances during pregnancy most frequently mentioned miscellaneous and plant products (Table S2), in contrast with the apparent major cross‐cultural avoidance of meat in general food taboos (Navarrete & Fessler 2003; Simoons 1994; Maggiulli et al. 2022). Women who lived in households that rely on agriculture alone or on agriculture‐husbandry/fishery, hereafter referred to in combination as agriculturalists, declared major food avoidances for plant products and miscellaneous foods overall, contrasting with fisher women (Figures 1 and 2). The most frequently avoided plant products among agriculturalists were staples such as flour products and locally cultivated bananas and *haninkotrana* (a Malagasy word indicating rice‐substituting staple crops like cassava and corn, or snacks), while the few cultivated products mentioned by fishers were mostly nonstaples (Figure S1). Animal product avoidances were more common among fishers, with a majority of them relating to commonly consumed marine species such as octopus and crab, but also marine species not typically consumed, such as dolphin and moray (Figure S1). The daily exposure to fish consumption derived from the catch of the day can explain the presence of marine species among fishers' taboos, even if fishers tend to name fish as taboo less frequently than agriculturalists do with agricultural products. Conversely, the fact that pig was the most mentioned nonmarine food source avoided by fishers is likely to reflect it being generally a banned animal in the south of Madagascar (Poirier 1978), where fisher women were interviewed, and that such a taboo could be reinforced during pregnancy by *ombiasy,* the local witch‐doctors. The lack of avoidance of cultivated products by fisher women in the southwest coast aligns with the fact that the local dry soil makes this area the least suitable for agriculture in Madagascar and the most prone to droughts (Dorosh et al. 2003; Dostie et al. 1999). Despite commercial flows of cassava being substantially redirected to the south during drought years, it does not provide more than 28% of energy intake, while rice consumption contributes only 20%, less than half of that in the rest of the country (Dostie et al. 1999; Dostie et al. 2002; Dorosh et al. 2003).

Therefore, foods avoided during pregnancy in Madagascar tend to mirror the staples obtained by agriculture and fishery, namely the two main modes of food production for self‐consumption in Madagascar.

### 4.3 Hypothesis II, III, and Secondary Question A. Fear of Difficult Delivery Distinguishes Food Avoidances in Agricultural Societies in Association With Local and Imported Sources of Carbohydrates, and the Concern Probably Predated Modern Nutrition Transition

The fear of big babies and/or difficult delivery was significantly higher among agriculturalists relative to fishers, while the latter were more concerned with varied and nonphysiologic complications (Figures 3 and 4). The fear of difficult delivery was more likely to be attributed to plant and miscellaneous products compared to animal food (Figures 5 and 6), and generally to food with higher carbohydrate content (Figure 7). Such results mirror differences in antenatal food prohibitions reported previously across subsistence patterns and across cultures (Maggiulli et al. 2022).

Among fishers, the association of consuming commonly eaten fish with allergies, of crabs with the possibility of transferring their excessive salivation in infants, and of rarely consumed dolphin and moray with witchcraft (Table 2), suggests that taboos may sometimes be driven by symbolic or supernatural motivations. Yet they may ultimately function to protect mothers and offspring from some actual physiological dangers. Fishers' taboos may also have some similarity with food aversions and disgust towards marine species experienced by fishers in the Fijian islands, which could have evolved to protect the mother and fetus from pathogens (McKerracher et al. 2016; Mckerracher et al. 2015; Placek and Hagen 2015; Placek et al. 2017). However, taboos explained by nausea and disgust in this study are rarely cited and not evenly spread across subsistence patterns. Therefore, while both cultural norms and food aversions have been described as adaptations to protect the mother and fetus from risks derived from changing physiological and nutritional requirements during pregnancy (Placek and Hagen 2015; Placek et al. 2017), we found that the two do not necessarily overlap, at least not relative to nonmarine species.

Our results do not allow us to explore the relation between food taboos, symbolism, and food aversions further; however, this could be addressed by future studies. Conversely, our results more strongly support the assumption that foods with a higher glycemic index are more likely to be associated with difficult delivery because of their major impact on fetal growth, and thus they are avoided through cultural norms among groups that are major producers and consumers of them, as previously observed in a cross‐cultural analysis (Maggiulli et al. 2022).

In this study, plant and miscellaneous products associated with difficult delivery are locally grown foods (bananas, peanuts, *haninkotrana*, gourds) or imported ones (wheat flour products and sugary foods) (Table 2). Wheat products, imported since the late nineteenth century and never widely produced in Madagascar (Johnston 1959; Randrianaivoarivony and Randriantsalama 1994), are less common in the Malagasy diet compared to rice, despite fried battered foods, noodles, bread, and baked sweets being appreciated as snacks or rice substitutes. Therefore, it is worth questioning whether the tendency to avoid carbohydrate sources to ease delivery followed the nutritional transitions stemming from colonialism and globalized markets (Ulijaszek et al. 2012) and was then extended to local items, or whether it existed prior to colonialism and was subsequently extended to imported goods. There is some evidence that the latter case is plausible. Acknowledging concerns about the reliability of old colonial ethnography, the traditional practice to prohibit bananas during pregnancy to avoid a big baby hindered colonial attempts to impose French standards of maternal healthcare (Decary 1930). While foreign wheat was not appreciated in Madagascar, local bananas, tubers, corn, gourds, nuts, and imported potatoes were largely valued (Phelps 1883) and their avoidance to avert large babies as a traditional practice is cited by Ravaoarisoa, Randriamanantsaina, et al. (2018) and Pourette et al. (2018).

General conceptions about pregnancy and food proscriptions also indicate a rooted fear of difficult delivery due to infant size in Madagascar. In the present study, one hospital staff member reported that A woman must not *love to eat* during pregnancy is a common Malagasy belief; the similar proverb Pregnant women who eats all that she finds: she will have a difficult day [at delivery], she will deliver in pain was recorded by Veyrières and Meritens (1967). Nonnutritional taboos during pregnancy informally explored during the interviews advised against sitting on the doorstep of houses to avoid obstructed labor. According to some respondents, the fattening effect of tabooed food on the fetus can be reversed by drinking a decoction of a leafy vegetable (*anantsipolitra*), thought to produce small babies. The consumption of other types of herbal infusions to avoid big babies is cited by Nguyen (2015).

It is noteworthy that rice, which provides the primary contribution to carbohydrate intake and has high cultural value in Madagascar, was considered to promote difficult delivery only by one respondent and never linked with other types of complications (Table 2). Perhaps it is exactly the nutritional and cultural importance of rice that buffers any actual or perceived association to weight gain during pregnancy. By contrast, the avoidance of tubers (*haninkotrana*) to avert large babies may have spread as an easy strategy to accomplish the social pressure to eat down, given the lower nutritional and cultural value of tubers (Ravaoarisoa, Rakotonirina, et al. 2018). Tubers may have represented the primary staple since the first human settlements on the island around the sixth century until the thirteenth/fourteenth centuries, when Indonesian groups brought rice farming together with rice consumption as a prerogative of the new aristocrats and the downgrading of tubers to a secondary staple (Beaujard 2011; Pierron et al. 2017; Ahmadi et al. 2021; Crowther et al. 2016). Based on rice or tubers, the historical centrality of carbohydrate in the Malagasy diet seems to be undebated, to the point that current patterns have been described as non‐diversified and over‐centered on rice and cassava (Ravaoarisoa, Rakotonirina, et al. 2018). The combination of heavy dependence on carbohydrates and a relatively recent and isolated genetic and phenotypic pool (Pierron et al. 2017) may have impacted pregnancy outcomes and favored concerns over large infant size. Such concerns arose plausibly before the modern nutritional transition and later addressed novel foods.

### 4.4 Secondary Question B. Fear of Large Babies and Difficult Delivery Drives Food Avoidance Across Regions: Evidence That Maternal Dimensions, Rather Than High Birth Weight, May Determine Obstructed Labor

In the agricultural highlands, women tend to avoid cultivated products for the fear of difficult delivery, and the proportion of women who follow antenatal food restrictions is highest in this region (Figure 8). This suggests that the association of carbohydrate staples with the fear of difficult delivery has become the primary driver of antenatal dietary restrictions in Madagascar: the number of avoiders is higher where the availability of plant products and foods perceived as fattening for the baby is higher, while a looser adherence to dietary restrictions is observed where the availability of potential fattening food, and thus the concern over obstructed labor, is lower.

The assessment of the actual rate of obstructed labor due to large infant size goes beyond the scope and potential of our study. However, we have reason to think that the actual size of the baby is not the main trigger of obstructed labor in Madagascar, and future research should also focus on maternal features, particularly in high carbohydrate‐consuming regions. In fact, self‐reported birth weights (Supporting Information) fall within the low and normal ranges regardless of subsistence pattern, as recorded in other clinical studies in Madagascar (Ratsiatosika et al. 2018, 2019; Ravaoarisoa et al. 2020; Vice Primatrue Charge de Sante Publique et al. 2010). Interestingly, agricultural women in the highlands are more likely to have height < 150 cm compared with other regions (WFP and UNICEF 2011), and some agricultural mothers referred to *Having a narrow pelvis* as a personal fear about delivery (Table S5) and to *having a narrow uterus* and *Difficult passage of the baby* as self‐reported experienced causes of difficult delivery (Table S6). In contrast, the percentage of maternal height < 150 cm in the south, the dry land where the surveyed fisher communities are based and access to carbohydrate is limited, is the lowest in Madagascar (Dostie et al. 1999; Dostie et al. 2002; Dorosh et al. 2003; WFP and UNICEF 2011). Considering the high rates of dystocia registered in agricultural areas (Vice Primatrue Charge de Sante Publique et al. 2010), it is likely that what is considered a big baby is a relative issue, and that a baby with a birth weight that might even seem on the low side (< 3 kg) by international criteria might still lead to a difficult delivery for an individual mother. This may be particularly true for mothers with short stature in regions where carbohydrate sources are largely available, as there is growing evidence that childbirth complications are likely to be exacerbated by (i) maternal short stature and reduced pelvic dimensions which are associated with growth faltering in early life or adolescence (Adadevoh et al. 1989; Shirley et al. 2020); (ii) the exposure to adequate or more than adequate carbohydrate intake in adulthood, because higher BMI in adult short mothers results in relatively higher fetal size (Wells et al. 2012; Wells et al. 2021; Wells et al. 2018; McCuskee et al. 2018; Hardenbergh 1997). This potentially explains why percentages of low birth weight in Madagascar are the lowest in agricultural regions and increase in the south where limited access to carbohydrate fosters nutritional stress (WFP and UNICEF 2011; Dostie et al. 1999; Dostie et al. 2002; Dorosh et al. 2003), and hints that a cascade of environmental, economic, and health factors influence different dietary behaviors around pregnancy. In line with recent nuanced interpretations of the obstetrical dilemma hypothesis, which embrace multiple factors and trade‐offs, as well as local manifestations and plasticity (Wells et al. 2012; Haeusler et al. 2021; Grunstra et al. 2023; Warrener 2023), our results are relevant to highlighting a local and nuanced situation, i.e., that Malagasy culture demonstrates a notable concern over difficult delivery and there is a plausible interaction of this concern with current growth and body composition profiles and diet.

### 4.5 Secondary Question C. Concern on Obstructed Labor as Convergence Point Between Institutional and Non‐Institutional Medical Knowledge

Our study confirms that antenatal behavior in Madagascar is influenced by an interplay between community level and institutional medical advice (Pourette et al. 2018; Andrianantoandro et al. 2021). Food avoidances explained by non‐physiologic complications are more commonly transmitted by non‐institutional sources, while those explained by varied physiologic danger and difficult delivery are more evenly transmitted by both sources. However, the two sources agree on which specific food to avoid only when it is related to difficult delivery (Figure S3). According to a hospital staff member interviewed in the highlands, people from rural areas perceive the medical advice to reduce the consumption of some aliments as a prohibition because they are accustomed to thinking in a taboo perspective. Therefore, it is possible that pre‐existing community level knowledge about eating down is unintentionally exacerbated by healthcare staff because mothers selectively retain the recommendations that resonate with taboos to avoid large babies, as highlighted by Pourette et al. (2018).

However, it is worth noting that the most cited medical advice to avert difficult delivery is to avoid salt, and that this is widely known across the sample (Table 2 and Table S8). This result does not fit the convergence of community knowledge with medical validity, which may be traced to the avoidance of fattening foods. The major concern over the consumption of salt was the development of *albuminuria*, a condition that leads to swelling of the legs and the uterine cervix, which would obstruct the passage of the infant during delivery. Two doctors affirmed that this guideline originated from annual training offered to medical staff by the public service or NGOs, which sporadically focus on maternal health. To our knowledge, no current medical literature confirms a causal relation between salt consumption and childbirth complications. Hints of traditional associations of salt consumption with difficult delivery and even fattening potential for cattle can be found in Poirier (1978), Pourette et al. (2018), Nguyen (2015), and Ellis (1839). In contrast, French colonial doctors associated salt with *albuminuria* (an increased content of albumin in the urine that accompanies swellings) and dystocia with uterine cervix rigidity (France Ministère des colonies 1898; Lecorché and Talamon 1888).

**FIGURE 9:**
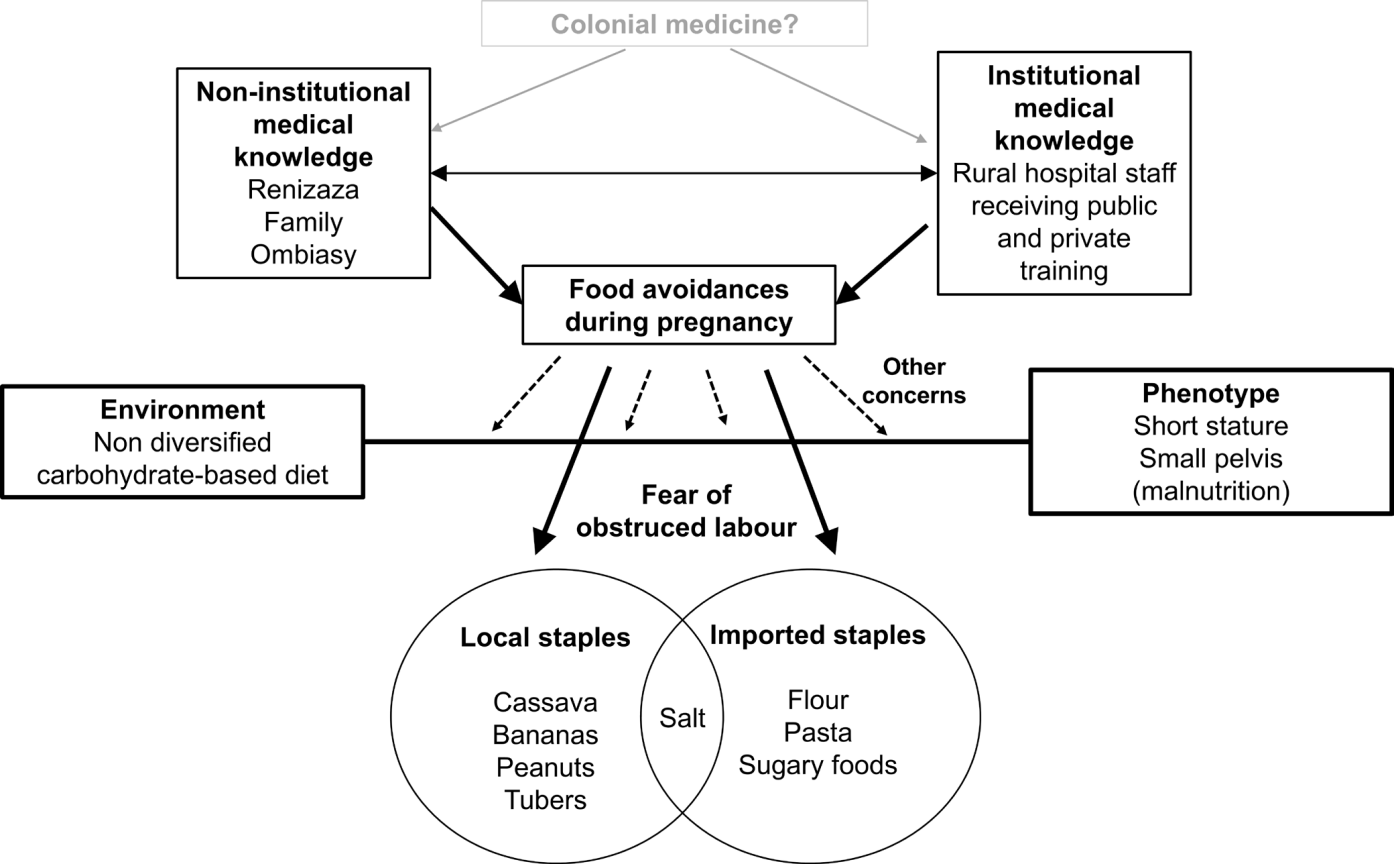
Cultural and biological factors, which shape antenatal dietary behavior in Madagascar. Antenatal dietary choices are influenced by two main groups of cultural points of reference: Traditional birth attenders (reninjaza), witch‐doctors (ombiasy), and family members who embody intergenerational transmission of non‐institutional knowledge; hospital staff established in rural areas, who are trained in public and/or private institutions. Noninstitutional medical knowledge responds to changing environmental and cultural scenarios; therefore, taboos are likely to embrace institutional guidelines and include imported foods over time. On the other hand, in the absence of up‐to‐date training or ongoing dialogue with communities, rural health care institutions might embrace or unintentionally reinforce non‐institutional medical knowledge. At a speculative level, residual advice pertaining to obsolete colonial medicine might persist in both noninstitutional and institutional knowledge around pregnancy. Among varied traditional and medical advice on antenatal dietary restrictions, the most common prohibitions target local and imported carbohydrate staples for the fear of obstructed labor. This concern may be stronger than others because phenotypic traits such as short stature and small pelvis maintained in the population by malnutrition, combined with a non‐diversified, carbohydrate‐rich diet, may produce ideal conditions for obstructed labor, which commonly occurs in rural areas.

We speculate that colonial medical information may have blended with local knowledge, possibly leading to the spread of the term *albumina* after *albuminuria* to indicate swelling not only of the legs but also of the uterus. The ability of Malagasy traditional practices to absorb new elements rather than capitulate to western medicine (Campbell 1992; Anderson 2017; Paillard 1987) and their fluidity over time (Golden and Comaroff 2015) has already been addressed in the literature. We further argue that the fluidity of what is valued as modern medical advice, in Madagascar as in other contexts, should not be overlooked. The fact that women tend to perceive institutional healthcare centers as safe and modern, despite these centers being poorly equipped and updated (Pourette et al. 2018), may reinforce trust in obsolete medical standards. Dietary behavior during pregnancy may then be shaped by a delicate interplay between environment, phenotypic features, and historical events, as illustrated in *Figure* *9*. However, the fact that the bonding agent between community‐level and institutionally transmitted practices is the concern over obstructed labor highlights the need to understand its determinants in Madagascar.

## 5 Limitations

The present study has the following limitations: self‐reported weight at birth of babies recalled by mothers may be inaccurate, so the magnitude of differences in birth weight across groups may be obscured; this is a preliminary study on the most common food taboos during pregnancy and reasons, with the aim of detecting explicit reference to physiologic dangers; therefore, the impact of symbolism or the supernatural sphere on women's perception of danger could not be explored in depth; this study did not collect clinical data on the respondents, preventing us from investigating the physiological determinants or incidence of difficult delivery. We hope that future work will deepen understanding of the emic nuances that drive food avoidance during pregnancy in Madagascar, as well as monitor both food habits and clinical development of pregnant women to shed light on obstructed labor and its relation to diet and culture.

## 6 Conclusion

We conducted the first cross‐regional survey on food taboos during pregnancy in Madagascar, and we compared the data across subsistence patterns. We observed that agriculturalists tend to avoid more plant and processed foods to prevent having a large baby and a difficult delivery compared with fishers, who avoid animal foods for nuanced reasons within the physiological but also symbolic sphere. The fear of difficult delivery mirrors the high incidence of dystocia in rural areas. On the basis of clinical data available in the literature, we propose that the combination of maternal features and a diet over‐centered on carbohydrates, rather than infant size, may lead to obstructed labor. Local phenotype and diet may therefore influence cultural norms on dietary behavior during pregnancy. We highlighted that future studies should investigate determinants of obstructed labor in Madagascar as well as the impact of colonial culture and novel foods on the perception of risk during pregnancy.

## Author Contributions

**Ornella Maggiulli:** conceptualization (lead), data curation (lead), formal analysis (lead), funding acquisition (lead), investigation (lead), methodology (lead), writing – original draft (lead). **Jean Freddy Ranaivoarisoa:** investigation (supporting), project administration (supporting), resources (supporting), supervision (supporting), writing – review and editing (supporting). **Hary Raliarison:** investigation (supporting), methodology (supporting), project administration (supporting), resources (supporting), supervision (supporting). **Mikanto Rabearison:** data curation (supporting), investigation (supporting), methodology (supporting), project administration (supporting). **Fifaliana Andriamanantena:** data curation (supporting), investigation (supporting), methodology (supporting), project administration (supporting). **Sabrinah Raherimamonjy:** data curation (supporting), investigation (supporting), methodology (supporting), project administration (supporting). **Jay T. Stock:** conceptualization (supporting), formal analysis (supporting), resources (supporting), supervision (supporting), writing – review and editing (supporting). **Jonathan C. K. Wells:** conceptualization (supporting), formal analysis (supporting), methodology (supporting), resources (supporting), supervision (supporting), writing – review and editing (supporting).

## Supporting information


**Figure S1.** Frequencies of plant products (a), animal products (b), and miscellanoeus (c) avoided by agriculturalists (intended as respondents practicing agriculture alone or combined with animal husbandry or fishery, white) and fishers (black).

**Figure S2.** Boxplot with jitter points that shows the visualize the distribution of weight at birth across groups.

**Figure S3.** Heatmap showing normalized frequencies of each food taboo (*y* axis) within clusters of reasons (“*b*” = large infants; “*v*” = varied physiologic reasons; “*np*” = non physiologic reasons) provided by medical staff (“*H*”) and non‐medical staff (“*T*”) (*x* axis). Lower values are in light yellow shades, and high values in orange to dark red colors. Since the original frequency values (tab 11) were normalized and scaled within columns, darker shades reflect higher frequencies within the corresponding column.

**Table S1.** List of variables extracted by the semi‐structured interviews.

**Table S2.** Frequency and percentage of foods avoided during pregnancy mentioned by Malagasy women practicing agriculture, agriculture‐husbandry, and fishey, grouped by food type.

**Table S3.** Frequency and percentage of all reasons to avoid foods during pregnancy mentioned in the interviews to Malagasy women, grouped by focus of the avoidance.

**Table S4.** Frequency and percentages of self‐reported birthweight (kg) of offspring of 207 respondents, divided by birthweight categories. Birthweights refer to the mean value if more than one weight was reported. Self‐reported cases of difficult delivery are indicated in correspondence of the weight of the infant.

**Table S5.** Personal fears about delivery of 87 respondents.

**Table S6.** Pregnancy complications reported by 38 respondents.

**Table S7.** PCA loadings (a) and scores (b).

**Table S8.** Foods avoided during pregnancy with specific reasons grouped by reason focus and source of the advice (institutional and non‐institutional healthcare staff). Frequencies for reasons greater that one are specified in parenthesis.

**Table S9.** Frequencies of food avoidances that were advised to avoid not physiologic dangers, varied physiologic dangers, and large infants, from non‐insitutional (NI) and institutional staff (I), respectively.

**Data S1.** Dataset

## Data Availability

The data that supports the findings of this study are available in the Supporting Information of this article.
