# Supplementary material for: Antenatal Food Avoidances in Madagascar Suggest an Evolutionary Link Between Subsistence Patterns, Carbohydrate Consumption, and Determinants of Obstructed Labor
Source: Am J Biol Anthropol. 2025 Mar 19;186(3):e70029. doi: 10.1002/ajpa.70029 (PMC11923398; doi:10.1002/ajpa.70029)
Supplement: Supplementary file 1 — Figure S1. Frequencies of plant products (a), animal products (b), and miscellanoeus (c) avoided by agriculturalists (intended as respondents practicing agriculture alone or combined with animal husbandry or fishery, white) and fishers (black). [file AJPA-186-e70029-s008.pdf]

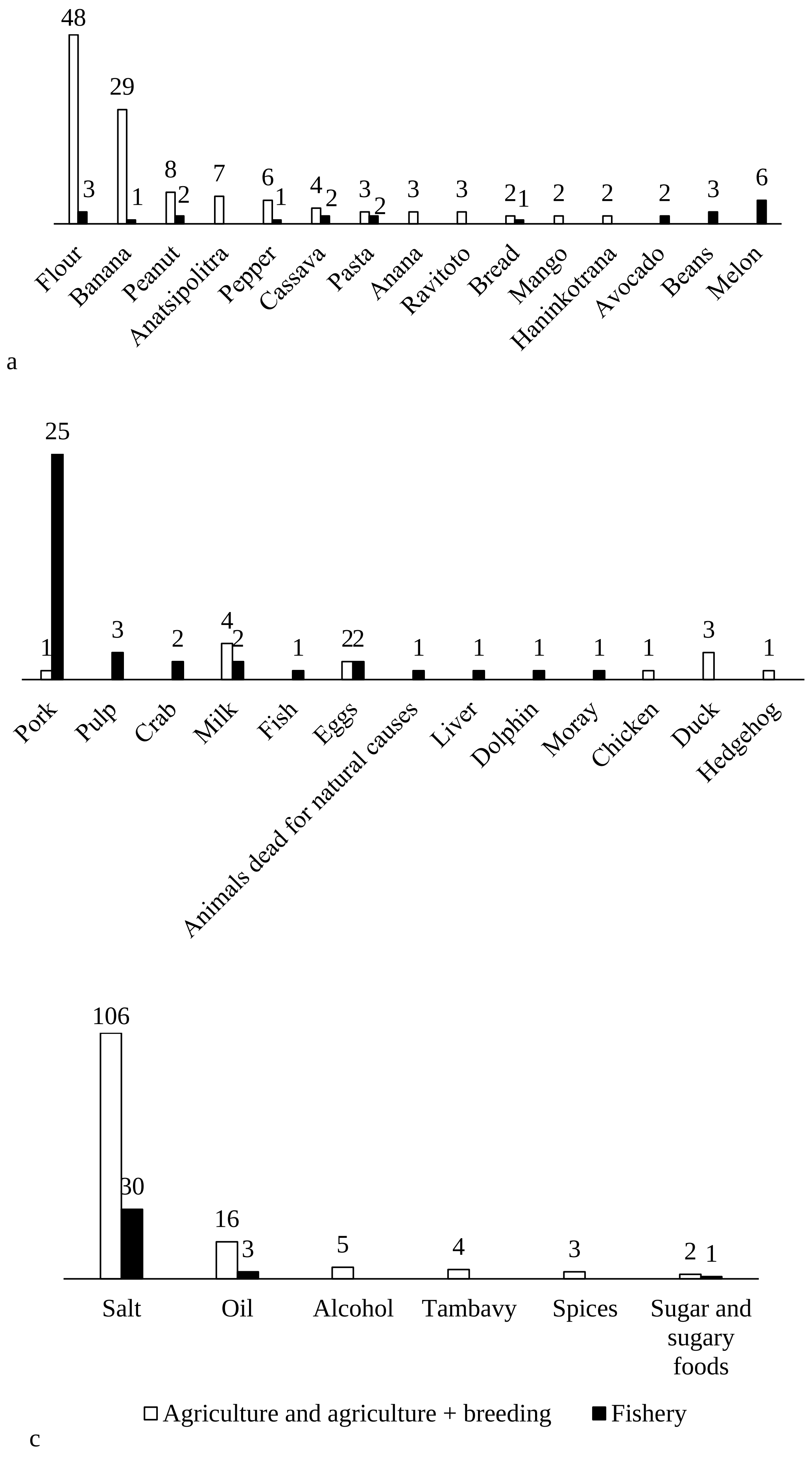

Figure 1 Frequencies of plant products (a), animal products (b), and miscellanoeus (c) avoided by agriculturalists (intended as respondents practicing agriculture alone or combined with animal husbandry or fishery, white) and fishers (black)
