# Supplementary material for: Antenatal Food Avoidances in Madagascar Suggest an Evolutionary Link Between Subsistence Patterns, Carbohydrate Consumption, and Determinants of Obstructed Labor
Source: Am J Biol Anthropol. 2025 Mar 19;186(3):e70029. doi: 10.1002/ajpa.70029 (PMC11923398; doi:10.1002/ajpa.70029)
Supplement: Supplementary file 2 — Figure S2. Boxplot with jitter points that shows the visualize the distribution of weight at birth across groups. [file AJPA-186-e70029-s003.pdf]

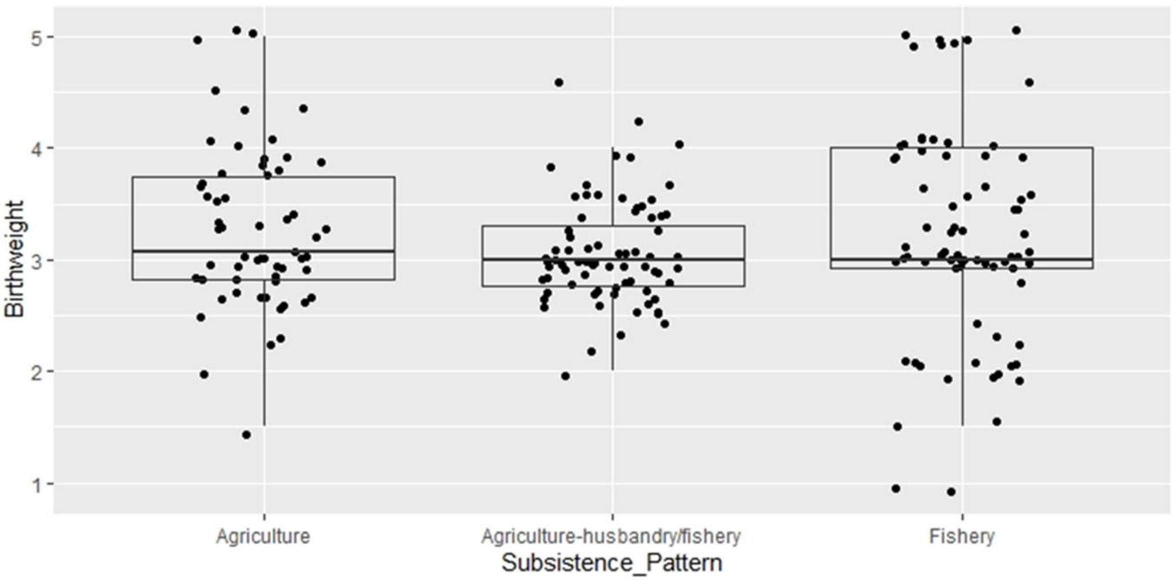

Figure 2 boxplot with jitter points that shows the visualize the distribution of weight at birth across groups
