# Supplementary material for: Antenatal Food Avoidances in Madagascar Suggest an Evolutionary Link Between Subsistence Patterns, Carbohydrate Consumption, and Determinants of Obstructed Labor
Source: Am J Biol Anthropol. 2025 Mar 19;186(3):e70029. doi: 10.1002/ajpa.70029 (PMC11923398; doi:10.1002/ajpa.70029)
Supplement: Supplementary file 3 — Figure S3. Heatmap showing normalized frequencies of each food taboo (y axis) within clusters of reasons (“b” = large infants; “v” = varied physiologic reasons; “np” = non physiologic reasons) provided by medical staff (“H”) and non‐medical staff (“T”) (x axis). Lower values are in light yellow shades, and high values in orange to dark red colors. Since the original frequency values (tab 11) were normalized and scaled within columns, darker shades reflect higher frequencies within the corresponding column. [file AJPA-186-e70029-s004.pdf]

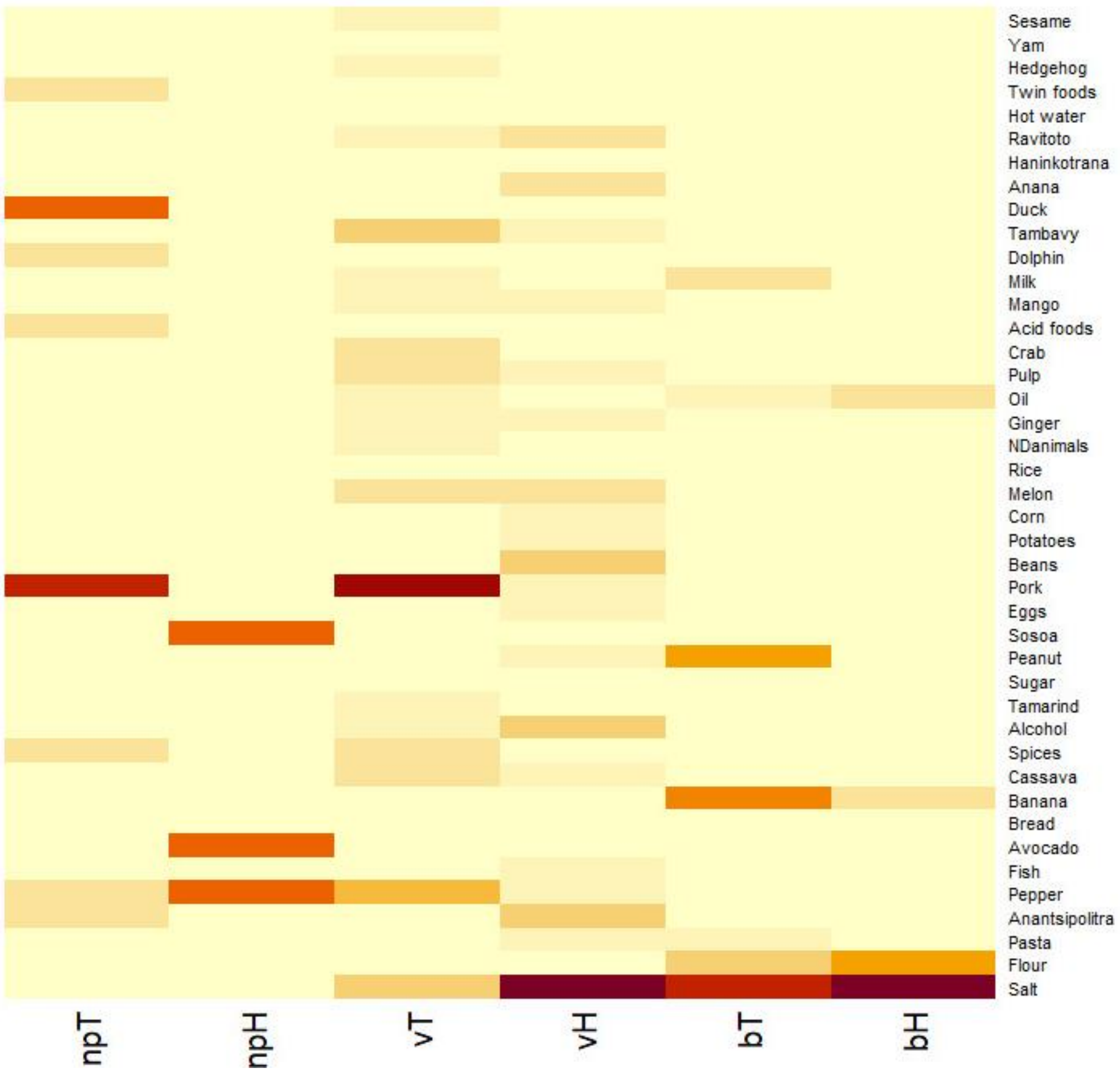

Figure 3 Heatmap showing normalized frequencies of each food taboo (Y axis) within clusters of reasons ("b" = large infants; "v" = varied physiologic reasons; "np" = non physiologic reasons) provided by medical staff ("H") and non-medical staff ("T") (X axis). Lower values are in light yellow shades, and high values in orange to dark red colors. Since the original frequency values (Table 11) were normalized and scaled within columns, darker shades reflect higher frequencies within the corresponding column.
