# Supplementary material for: Antenatal Food Avoidances in Madagascar Suggest an Evolutionary Link Between Subsistence Patterns, Carbohydrate Consumption, and Determinants of Obstructed Labor
Source: Am J Biol Anthropol. 2025 Mar 19;186(3):e70029. doi: 10.1002/ajpa.70029 (PMC11923398; doi:10.1002/ajpa.70029)
Supplement: Supplementary file 4 — Table S1. List of variables extracted by the semi‐structured interviews. [file AJPA-186-e70029-s005.pdf]

Table 1 List of variables extracted by the semi-structured interviews.

Variable name	Type	Item
Respondent	Nominal	String (each respondent was identified by the name of location followed by a progressive number)
Location	Nominal	Numeric (1 = Highlands, 2 = Southwest coast, 3 = Marovoay, 4 = Maroantsetra)
Subsistence pattern	Nominal	Numeric (1 = Agriculture, 2 = Agriculture-husbandry, 3 = Fishery)
Food_name (1…n)	Nominal	Numeric (1 = Mentioned, 2 = Not mentioned
Food_type (1…n)	Nominal	Numeric (1 = Animal product, 2 = Plant product, 3 = Miscellaneous)
Reason_for_name of food (1…n)	Nominal	String (reason for the avoidance as provided by the respondent)
Reason_focus	Nominal	Defined *a posteriori*: Numeric (1 = Big baby and/or difficult delivery, 2= Varied physiologic complications, 3 = Non-physiologic complications)
Food_teller	Nominal	Numeric (1 = Traditional, 2 = Hospital staff)
Personal fears about delivery	Nominal	String (self-reported fears about delivery)
Complications	Nominal	String (self-reported complications experienced during pregnancy)
Birthweight	Scale	Numeric (self-reported birthweights of infants; the mean value was calculated if more than one weight was provided)
N of daily meals before pregnancy	Scale	Numeric
N of daily meals during pregnancy	Scale	Numeric
Perception of the pregnancy experience	Ordinal	Numeric (0 = Negative, 1= Neutral, 2 = Positive)
