# Supplementary material for: Antenatal Food Avoidances in Madagascar Suggest an Evolutionary Link Between Subsistence Patterns, Carbohydrate Consumption, and Determinants of Obstructed Labor
Source: Am J Biol Anthropol. 2025 Mar 19;186(3):e70029. doi: 10.1002/ajpa.70029 (PMC11923398; doi:10.1002/ajpa.70029)
Supplement: Supplementary file 5 — Table S2. Frequency and percentage of foods avoided during pregnancy mentioned by Malagasy women practicing agriculture, agriculture‐husbandry, and fishey, grouped by food type. [file AJPA-186-e70029-s002.pdf]

Table 2 Frequency and percentage of foods avoided during pregnancy mentioned by Malagasy women practicing agriculture, agriculture-husbandry and fishey, grouped by food type.

Animal products			Plant products			Miscellaneous		
	N	%		*N*	%		*N*	%
Pig	26	51	Flour	51	34	Salt	136	78
Milk	6	12	Banana	30	20	Oil	19	11
Eggs	4	8	Peanut	10	7	Alcohol	5	3
Octopus	3	6	Anatsipolitra (herb)	7	5	Tambavy (herbal infusion)	4	2
Duck	3	6	Pepper	7	5	Spices	3	2
Crab	2	4	Melon	6	4	Sugar and sugary	3	2
Fish	1	2	Pasta	5	3	Acid foods	1	1
Chicken	1	2	Cassava	6	4	Hot water	1	1
Animals dead for natural causes	1	2	Bread	3	2	Twin foods	1	1
Liver	1	2	Beans	3	2	Foods that make baby bigger	1	1
Dolphin	1	2	Anana (leafy vegetable)	3	2	**Total**	174	100
Moray	1	2	Ravitoto (cassava leaves)	3	2			
Hedgehog	1	2	Avocado	2	1			
**Total**	51	100	Sweet potatoes	2	1			
			Rice	2	1			
			Ginger	2	1			
			Mango	2	1			
			Haninkotrana (tubers)	2	1			
			Tamarind	1	1			
			Sosoa (wet rice)	1	1			
			Corn	1	1			
			Yam	1	1			
			Apango (rice crust)	1	1			
			Sesame	1	1			
			**Total**	152	100			
