# Supplementary material for: Antenatal Food Avoidances in Madagascar Suggest an Evolutionary Link Between Subsistence Patterns, Carbohydrate Consumption, and Determinants of Obstructed Labor
Source: Am J Biol Anthropol. 2025 Mar 19;186(3):e70029. doi: 10.1002/ajpa.70029 (PMC11923398; doi:10.1002/ajpa.70029)
Supplement: Supplementary file 6 — Table S3. Frequency and percentage of all reasons to avoid foods during pregnancy mentioned in the interviews to Malagasy women, grouped by focus of the avoidance. [file AJPA-186-e70029-s013.pdf]

Table 3 Frequency and percentage of all reasons to avoid foods during pregnancy mentioned in the interviews to Malagasy women, grouped by focus of the avoidance.

**Big baby and/or difficult delivery**			**Varied physiologic complications**			**Non-physiologic complications**		
	N	*%*		*N*	*%*		*N*	*%*
Big baby	191	63.9	Farasisa	24	27.9	Trembling during delivery	4	20
Albumina	98	32.8	Too thin	12	14	Witchcraft	3	15
Too much oil for the baby and difficult delivery	10	3.3	Sickness of child or mother	10	11.6	Crazy baby	3	15
**Total**	299	100	Abortion	10	11.6	Webbed fingers	3	15
			Vomit or nausea or disgust	6	7	Its oil contaminates the baby	2	10
			Bone decalcification	4	4.6	Excessive salivation for the baby	2	10
			Handicap	4	4.6	Dirty baby	1	5
			Stomach-ache	4	4.6	Twin organs	1	5
			Itchy for the baby	3	3.5	Soft baby	1	5
			Haemorrhage	2	2.3		20	100
			Yellow fever	2	2.3			
			Allergy	1	1.2			
			Diarrhoea for the baby	1	1.2			
			Skin problems	1	1.2			
			Boils	1	1.2			
			Spot on the back	1	1.2			
			**Total**	86	100			
