# Supplementary material for: Antenatal Food Avoidances in Madagascar Suggest an Evolutionary Link Between Subsistence Patterns, Carbohydrate Consumption, and Determinants of Obstructed Labor
Source: Am J Biol Anthropol. 2025 Mar 19;186(3):e70029. doi: 10.1002/ajpa.70029 (PMC11923398; doi:10.1002/ajpa.70029)
Supplement: Supplementary file 7 — Table S4. Frequency and percentages of self‐reported birthweight (kg) of offspring of 207 respondents, divided by birthweight categories. Birthweights refer to the mean value if more than one weight was reported. Self‐reported cases of difficult delivery are indicated in correspondence of the weight of the infant. [file AJPA-186-e70029-s012.pdf]

Table 4 Frequency and percentages of self-reported birthweight (kg) of offspring of 207 respondents, divided by birthweight categories. Birthweights refer to the mean value if more than one weight was reported. Self-reported cases of difficult delivery are indicated in correspondence of the weight of the infant.

	Birthweight (kg)	N	%	Self-reported cases of difficult delivery
**Low (< 2.5 kg)**	**1**	**2**	**1**	
	**1.5**	**3**	**1.4**	**1**
	**2**	**11**	**5.3**	
	**2.1**	**2**	**1**	
	**2.2**	**1**	**0.5**	
	**2.3**	**3**	**1.4**	**1**
	**2.4**	**1**	**0.5**	
			**Tot. 11.1**	
**Normal (2.5-3.9 kg)**	2.5	6	2.9	
	2.55	1	0.5	
	2.57	1	0.5	
	2.6	6	2.9	
	2.63	1	0.5	
	2.65	1	0.5	1
	2.67	1	0.5	
	2.69	1	0.5	
	2.7	4	1.9	
	2.75	5	2.4	1
	2.8	7	3.3	
	2.85	1	0.5	
	2.9	10	4.8	1
	2.93	1	0.5	
	2.95	1	0.5	
	3	45	21.5	2
	3.07	1	0.5	
	3.1	3	1.4	
	3.15	2	1	
	3.2	6	2.9	
	3.25	3	1.4	
	3.27	1	0.5	
	3.3	6	2.9	2
	3.4	4	1.9	2
	3.5	15	7.2	1
	3.55	1	0.5	
	3.6	1	0.5	
	3.65	1	0.5	
	3.67	1	0.5	
	3.7	1	0.5	
	3.75	6	2.9	1
	3.77	1	0.5	

	3.8	1	0.5	
	3.87	1	0.5	
	3.9	2	1	
	4	19	9.1	1
			**Tot. 80.9**	
	4.3	2	1	
**High (macrosomia, > 3.9)**	4.4	1	0.5	1
	4.5	3	1.4	
	5	10	4.8	2
			**Tot. 7.7**	
