# Supplementary material for: Antenatal Food Avoidances in Madagascar Suggest an Evolutionary Link Between Subsistence Patterns, Carbohydrate Consumption, and Determinants of Obstructed Labor
Source: Am J Biol Anthropol. 2025 Mar 19;186(3):e70029. doi: 10.1002/ajpa.70029 (PMC11923398; doi:10.1002/ajpa.70029)
Supplement: Supplementary file 8 — Table S5. Personal fears about delivery of 87 respondents. [file AJPA-186-e70029-s001.pdf]

Table 5 Personal fears about delivery of 87 respondents.

Personal fears about delivery of 87 respondents	*N*	*%*	*Detail*
Difficult or late delivery	54	63	Narrow pelvis (3), Albumina (3), Difficult delivery and caesarean section (3), Difficult passage of the baby (2), Difficult delivery and death of baby at birth (2), Difficult delivery for hydrocephalus, Difficult delivery due to malposition, Narrow uterine cervix, Not having strength to pull the baby out, Baby does not pass, Difficult delivery for big baby
Malposition	13	14	
Baby born dead	10	11	
Haemorrhage	10	11	
Umbilical cord tied around the neck	8	9	
Nausea or vomit	2	2	
Preterm delivery	2	2	Being tired and deliver before time
Induced labour	2	2	Not being able to break waters, Tension
Labour pain, a lot of water during delivery	1	1	
Suffocated due to the spices	1	1	
A lot of water during delivery	1	1	
Dizziness	1	1	
Malformation	1	1	
No blood during delivery	1	1	
