# Supplementary material for: Antenatal Food Avoidances in Madagascar Suggest an Evolutionary Link Between Subsistence Patterns, Carbohydrate Consumption, and Determinants of Obstructed Labor
Source: Am J Biol Anthropol. 2025 Mar 19;186(3):e70029. doi: 10.1002/ajpa.70029 (PMC11923398; doi:10.1002/ajpa.70029)
Supplement: Supplementary file 9 — Table S6. Pregnancy complications reported by 38 respondents. [file AJPA-186-e70029-s011.pdf]

Table 6 Pregnancy complications reported by 38 respondents.

Pregnancy complications reported by 38 respondents	*N*	*%*	*Detail*
Difficult or late delivery	20	52.6	Albumina (2), Caesarean section (2), Difficult delivery and baby born dead, Difficult delivery and haemorrhage, narrow uterus cervix, Difficult passage of the baby, She has been cut during delivery
Abortion	6	15.8	
Haemorrhage	6	15.8	
Dizziness	5	13.2	
Tiredness	4	10.5	
Vomit or nausea	4	10.5	
Baby born dead	3	7.9	
Umbilical cord around the neck	3	7.9	
Headache	2	5.3	
A lot of water during delivery	2	5.3	
Asphyxia	1	2.6	
Cough	1	2.6	
No blood during delivery	1	2.6	
Malformation of the head, big head	1	2.6	
Lack of appetite	1	2.6	
Not enough milk	1	2.6	
