# Supplementary material for: Antenatal Food Avoidances in Madagascar Suggest an Evolutionary Link Between Subsistence Patterns, Carbohydrate Consumption, and Determinants of Obstructed Labor
Source: Am J Biol Anthropol. 2025 Mar 19;186(3):e70029. doi: 10.1002/ajpa.70029 (PMC11923398; doi:10.1002/ajpa.70029)
Supplement: Supplementary file 10 — Table S7. PCA loadings (a) and scores (b). [file AJPA-186-e70029-s009.pdf]

Table 7 PCA loadings (a) and scores (b)

**a)**	**PC1**	**PC2**
Avoiders	0.2405815	0.453480316
Difficult delivery	0.4075172	-0.109424273
Varied physiologic complications	0.1288342	0.537336729
Non-physiologic complications	-0.3620679	0.246853495
Agriculturalists	0.4145941	-0.006661288
Fishers	-0.4145941	0.006661288
Plant	0.155104	0.5266344
Animal	0.4145088	0.007653448
Miscellaneous	0.2978382	-0.393984025

**b)**	**PC1**	**PC2**
Highlands	1.4442486	2.4141229
Southwest	-3.5985440	0.1795227
Marovoay	1.1904199	-1.5035538
Maroantsetra	0.9638756	-1.0900918
