# Supplementary material for: Antenatal Food Avoidances in Madagascar Suggest an Evolutionary Link Between Subsistence Patterns, Carbohydrate Consumption, and Determinants of Obstructed Labor
Source: Am J Biol Anthropol. 2025 Mar 19;186(3):e70029. doi: 10.1002/ajpa.70029 (PMC11923398; doi:10.1002/ajpa.70029)
Supplement: Supplementary file 11 — Table S8. Foods avoided during pregnancy with specific reasons grouped by reason focus and source of the advice (institutional and non‐institutional healthcare staff). Frequencies for reasons greater that one are specified in parenthesis. [file AJPA-186-e70029-s010.pdf]

Table 8 Foods avoided during pregnancy with specific reasons grouped by reason focus and source of the advice (institutional and non-institutional healthcare staff). Frequencies for reasons greater that one are specified in parenthesis.

	**Big baby and/or difficult delivery**		**Varied physiologic complications**		**Non-physiologic complications**	
	Non insitutional staff	*Insitutional staff*	*Non insitutional staff*	*Insitutional staff*	*Non insitutional staff*	*Insitutional staff*
**Salt**	Albumina (14); Big baby	Albumina (67); Big baby (27)	Farasisa (3)	Farasisa (9); Sickness		Dirty baby
**Flour**	Big baby (5)	Big baby (40)				
**Pasta**	Big baby (2)	Big baby (2)		Nausea		
**Anantsipolitra**			Too thin baby	Too thin baby (3)		
**Pepper**			Farasisa (2); Abortion; Skin problems	Trembling; Farasisa	Trembling	
**Fish**				Allergy		
**Avocado**	Albumina					Dirty baby
**Bread**	Big baby	Big baby (2)				
**Banana**	Big baby (11)	Big baby (16)				
**Cassava**		Big baby	Bone decalcification	Too thin baby; Haemorrhage		
**Spices**			Farasisa (2)		Trembling	
**Alcohol**			Farasisa	Handicap; Farasisa (2)		
**Tamarind**			Too thin baby			
**Sugar**		Big baby (3)				
**Peanut**	Big baby; Too much oil for the baby and difficult delivery (8)			Sickness		
**Sosoa**						Soft baby
**Eggs**			Handicap			
**Pig**			Handicap (2); Sickness (5); Abortion (3)	Stomach-ache	Crazy baby (3)	
**Beans**			Sickness	Stomachache (2)		
**Potatoes**	Big babies			Nausea		
**Corn**				Haemorrhage		
**Melon**	Big baby		Sickness; Abortion	Nausea; Diarrhoea		
**Rice**		Big baby				
**Naturally dead animals**			Abortion			
**Ginger**			Sickness	Abortion		

**Oil**	Albumina*; Big baby	Albumina* (10); Big baby; Too much oil for the baby and difficult delivery	Boils			
**Octopus**			Itchy for the baby (2)	Itchy for the baby		
**Crab**					Excessive salivation (2)	
**Acid foods**					Trembling	
**Mango**			Spot on the back	Bone decalcification		
**Milk**	Big baby (4)		Abortion			
**Dolphin**					Witchcraft	
**Tambavy**			Farasisa**; Yellow fever (2)	Farasisa**		
**Duck**					Webbed fingers (3)	
**Anana**				Too thin baby; Stomachache		
**Haninkotrana**		Big baby (2)				
**Ravitoto**				Bone decalcification; Too thin		
**Hot water**	Big baby					
**Twin foods**					Twin organs	
**Hedgehog**			Abortion			
**Yam**	Big baby					
**Sesame**			Farasisa**			
**TOTAL**	54	182	39	47	13	3
