# Supplementary material for: Antenatal Food Avoidances in Madagascar Suggest an Evolutionary Link Between Subsistence Patterns, Carbohydrate Consumption, and Determinants of Obstructed Labor
Source: Am J Biol Anthropol. 2025 Mar 19;186(3):e70029. doi: 10.1002/ajpa.70029 (PMC11923398; doi:10.1002/ajpa.70029)
Supplement: Supplementary file 12 — Table S9. Frequencies of food avoidances that were advised to avoid not physiologic dangers, varied physiologic dangers, and large infants, from non‐insitutional (NI) and institutional staff (I), respectively. [file AJPA-186-e70029-s006.pdf]

Table 9 Frequencies of food avoidances that were advised to avoid not physiologic dangers, varied physiologic dangers, and large infants, from non-insitutional (NI) and institutional staff (I) respectively.

	**Not physiologic (NI)**	**Not physiologic (I)**	**Varied (NI)**	**Varied (I)**	**Big babies (NI)**	**Big babies (I)**
Salt	0	0	3	10	15	94
Flour	0	0	0	0	5	40
Pasta	0	0	0	1	2	2
Anantsipolitra	1	0	0	3	0	0
Pepper	1	1	4	1	0	0
Fish	0	0	0	1	0	0
Avocado	0	1	0	0	1	0
Bread	0	0	0	0	1	2
Banana	0	0	0	0	11	16
Cassava	0	0	2	1	0	2
Spices	1	0	2	0	0	0
Alcohol	0	0	1	3	0	0
Tamarind	0	0	1	0	0	0
Sugar	0	0	0	0	0	3
Peanut	0	0	0	1	9	0
Sosoa	0	1	0	0	0	0
Eggs	0	0	0	1	0	1
Pork	4	0	10	1	0	0
Beans	0	0	0	3	0	0
Potatoes	0	0	0	1	1	0
Corn	0	0	0	1	0	0
Melon	0	0	2	2	1	0
Rice	0	0	0	0	0	1
Naturally dead animals	0	0	1	0	0	0
Ginger	0	0	1	1	0	0
Oil	0	0	1	0	2	13
Pulp	0	0	2	1	0	0
Crab	0	0	2	0	0	0
Acid foods	1	0	0	0	0	0
Mango	0	0	1	1	0	0
Milk	0	0	1	0	4	0
Dolphin	1	0	0	0	0	0
Tambavy	0	0	3	1	0	0
Duck	3	0	0	0	0	0
Anana	0	0	0	2	0	0
Haninkotrana	0	0	0	0	0	2
Ravitoto	0	0	1	2	0	0
Hot water	0	0	0	0	1	0
Twin foods	1	0	0	0	0	0
Hedgehog	0	0	1	0	0	0

Yam	0	0	0	0	1	0
Sesame	0	0	1	0	0	0
